# Bioevaluation of Ranatuerin-2Pb from the Frog Skin Secretion of *Rana pipiens* and Its Truncated Analogues

**DOI:** 10.3390/biom9060249

**Published:** 2019-06-25

**Authors:** Xiaowei Zhou, Daning Shi, Ruimin Zhong, Zhuming Ye, Chengbang Ma, Mei Zhou, Xinping Xi, Lei Wang, Tianbao Chen, Hang Fai Kwok

**Affiliations:** 1 Department of Nutrition, Henry Fok School of Food Science and Engineering, Shaoguan University, Shaoguan 512005, China; xzhou06@qub.ac.uk (X.Z.); zhongrm9898@163.com (R.Z.); 2 Natural Drug Discovery Group, School of Pharmacy, Queen’s University, Belfast BT9 7BL, Northern Ireland, UK; dshi01@qub.ac.uk (D.S.); zye04@qub.ac.uk (Z.Y.); c.ma@qub.ac.uk (C.M.); m.zhou@qub.ac.uk (M.Z.); l.wang@qub.ac.uk (L.W.); t.chen@qub.ac.uk (T.C.); 3 School of Government, Peking University, No 114, The Leo KoGuan Building, Beijing 100871, China; 4 Institute of Translational Medicine, Faculty of Health Sciences, University of Macau, Avenida de Universidade, Taipa, Macau

**Keywords:** Ranatuerin, antimicrobial peptides, α-helix, membrane permeability, waxworm model

## Abstract

Antimicrobial peptides (AMPs) are considered as a promising agent to overcome the drug-resistance of bacteria. Large numbers of AMPs have been identified from the skin secretion of *Rana pipiens*, including brevinins, ranatuerins, temporins and esculentins. In this study, the cDNA precursor of a broad-spectrum antimicrobial peptide, ranatuerin-2Pb, was cloned and identified. Additionally, two truncated analogues, RPa and RPb, were synthesised to investigate the structure-activity relationship of ranatuerin-2Pb. RPa lost antimicrobial activity against *Candida albicans*, MRSA, *Enterococcus faecalis* and *Pseudomonas aeruginosa*, while RPb retained its broad-spectrum antimicrobial activity. Additionally, ranatuerin-2Pb, RPa and RPb demonstrated inhibition and eradication effects against *Staphylococcus aureus* biofilm. RPb showed a rapid bacterial killing manner via membrane permeabilization without damaging the cell membrane of erythrocytes. Moreover, RPb decreased the mortality of *S. aureus* infected *Galleria mellonella* larvae. Collectively, our results suggested that RPb may pave a novel way for natural antimicrobial drug design.

## 1. Introduction

In recent decades, the overuse of conventional antibiotics has aggravated the resistance of bacteria, and this has become a serious problem facing the world [1]. Facing this severe problem, the World Health Organization (WHO) delivered a global report in 2014, which mentioned that a large number of bacteria possess drug resistance. Consequently, developing a global intervention plan against antimicrobial resistance is a matter of high urgency [2]. Therefore, it is urgent and critical to develop a new class of antibiotics. Nowadays, it is widely recognised that AMPs could pave a novel way to fight against multidrug-resistance strains because of the rapid kill-time of strains through cell membrane disruption, but the development of AMP-resistant strains could be inevitable when applied to clinical pathogens [3,4,5,6].

Ranatuerin-2 peptides, were initially identified in the skin of *Lithobates catesbeianus* and have been found in most species of North American, Chinese and Japanese frogs [7,8]. These peptides contain a C-terminal cyclic domain; however, the primary structure of these peptides was poorly conserved with several residue deletions with only the cysteine residues invariant. For instance, two peptides, ranatuerin-2P and ranatuerin-2Pa, which were isolated in *Lithobates pipiens*, consisted of a C-terminal cyclic heptapeptide domain and a “Rana box”, respectively [9]. The primary structures of pranatuerin-2 peptides were poorly conserved, which results in huge characteristic differences of antimicrobial properties and haemolytic activities. For instance, ranatuerin-2CHb possessed broad-spectrum antimicrobial activity [10], however, ranatuerin-2ONa exhibited the growth inhibition ability against *Escherichia coli* (*E. coli*) and *Candida albicans* (*C. albicans*) but was inactive against *S. aureus* [9].

In this study, the precursor encoding cDNA sequence of ranatuerin-2Pb was determined by the “shotgun” cloning method. In addition, two analogues, RPa (removed RT) and RPb (recited 18 C-terminal amino acids and amidated) were designed to investigate structure–activity relationship of ranatuerin-2Pb. Both ranatuerin-2Pb and its analogues were synthesised and the purified replicates were subjected to bioactivity assessments. Afterwards, the in vivo study was performed by waxworm model to investigate the anti-*S. aureus* effect of RPb.

## 2. Materials and Methods 

### 2.1. Specimen Preparation and Secretion Harvesting

The specimens of the frog *Rana pipiens* were captured, settled and skin secretion was obtained from the dorsal skin as described previously [11]. The study was performed according to the guidelines in the UK Animal (Scientific Procedures) Act 1986, project license PPL 2694, issued by the Department of Health, Social Services and Public Safety, Northern Ireland. Procedures had been vetted by the IACUC of Queen’s University Belfast, and approved on 1st March, 2011. 

### 2.2. “Shotgun” Cloning of a Novel Ranatuerin-2Pb Encoded cDNA Library

The “shotgun” cloning was carried out as previously described [11]. A NUP primer and a sense degenerated primer (5′-GAWYYAYYHRAGCCYAAADATG-3′; W = A + T, Y = C + T, H = A + C + T, R = A + G, D = A + G + T) were subjected to 3′-RACE reaction, from which the products were cloned and subsequently sequenced. 

### 2.3. Peptide Synthesis

The novel peptide ranatuerin-2Pb (SFLTTVKKLVTNLAALAGTVIDTIKCKVTGGCRT) and two analogues RPa (SFLTTVKKLVTNLAALAGTVIDTIKCKVTGGC) and RPb (SFLTTVKKLVTNLAAL-NH_2_) were synthesized and purified by Gene Script Corporation (Nanjing, China) through solid-phase synthesis methods. The formation of disulfate bond was achieved by air-oxidation at room temperature for three days. The purity of the peptides was determined to be more than 95% by reverse-phase high-performance liquid chromatography (RP-HPLC). The peptides were further subjected to electrospray mass spectrometry to confirm their molecular mass. 

### 2.4. Conformation Study

Physiochemical properties of helical wheel projection of all peptides were predicted by Heliquest (http://heliquest.ipmc.cnrs.fr). The secondary structures of peptides were evaluated by circular dichroism (CD) spectroscopy (JASCO J-815, Essex, UK), as described previously [12]. The peptides solutions were prepared at 50 µM in deionised water, 50% 2,2,2-trifluoroethanol (TFE)/H_2_O and large unilamellar liposome vesicles (LUVs) solutions. 

DOPC, DOPE and DOPG (Avanti, Alabaster, AL, USA) were dissolved in chloroform to obtain stock solutions. The desired compositions were mixed and then dried under high vacuum evaporator until the lipid layer was formed at the bottom of the bottle. The resulting dispersions were extruded through a stack of two polycarbonate filters (200-nm pore size; Millipore Corp., Bedford, MA, USA) using a Liposofast low pressure homogeniser (Avestin, Ottawa, Canada) to obtain LUVs after five frozen-thaw cycles in 37°C. The concentration of total phosphorus was determined by ascorbic acid/ammonium molybdate method and 20 mM of liposomes was subjected to CD analysis. The estimated secondary structure was calculated by BESTSEL CD spectrum analysis tool [13]. 

### 2.5. Antimicrobial Activity 

Antimicrobial activity assay was evaluated by confirmation of minimal inhibitory concentration (MIC) using two strains of Gram-negative bacterium, *E. coli* (NCTC 10418) and *Pseudomonas aeruginosa (P. aeruginosa*, ATCC 27853), three strains of Gram-positive bacterium, *Staphylococcus aureus* (NCTC 10788), Meticillin-resistant *Staphylococcus aureus* (MRSA, ATCC 12493) and *Enterococcus faecalis* (*E. faecalis*, NCTC 12697) and the yeast, *C. albicans* (NCPF 1467), as indicated previously [14]. 5 × 10^5^ CFU/mL of bacterial suspension incubated with 2-fold dilution peptides (ranging from 1 µM to 256 µM) for 24 h at 37 °C. The antimicrobial activity of peptide was tested by monitoring the absorbance at 550nm using an ELISA plate reader (Biolise BioTek EL808, Winooski, VT, USA)

### 2.6. Antibiofilm Assays with Different Organisms

The antibiofilm ability of designed peptides was evaluated using two different organisms which were *S. aureus* (NCTC 10788), *E. coli* (NCTC 10418), as indicated previously [15]. For the MBIC assay, 5 × 105 CFU/mL bacterial cultures were incubated with 2-fold dilutions of peptides (ranging from 1 µM to 256 µM) for 24 h. Fresh medium and bacterial suspension as negative control and positive control, respectively. The absorbance values were measured at wavelength 595 nm using a Synergy HT plate reader (Biotech, Minneapolis, MN, USA). Experiments were done in triplicate and repeated twice independently.

### 2.7. Haemolysis Assay

The haemolysis assay was assessed using erythrocytes which were prepared from whole horse blood (TCS Biosciences Ltd. Buckingham, UK) as described previously [14]. PBS and 1% Triton-100 as negative and positive control, respectively. To further investigate the cell selectivity of the peptides, therapeutic index (TI) was calculated as HC_50_ divided by geometric mean of the peptide MICs against the relevant bacteria.

### 2.8. Membrane Permeability Assay

The membrane permeability assay was evaluated by SYTOX^TM^ green nucleic acid stain (Life Technologies) as described previously [16]. The nucleic acid dye bound to cell nucleus when the cell membrane was permeabilised. The fluorescent intensity was detected with a Synergy HT plate reader (Biotech, Minneapolis, MN, USA) by an excitation and emission wavelength of 485 and 528 nm, respectively. *S. aureus* treated with melittin (8 µM) for 1 h were used as positive controls.

### 2.9. MTT and Lactate Dehydrogenase (LDH) Cytotoxicity Assay

The cell viability of ranatuerin-2Pb, RPa and RPb (Concentration ranging from 10^-9^-10^-4^) was analysed using 5 human cancer cell lines which are H157 (ATCC CRL-5802), MCF-7 (ATCC HTB-22), U251MG (ECACC General Cell Collection: 09063001), PC-3 (ATCC CRL-1435) and MDA-MB-435s (ATCC HTB-129). Melittin (Concentration ranging from 10^−9^–10^−4^) regarded as positive control. The cell viability was assessed using MTT assay as described previously [14]. The LDH cytotoxicity assay was performed using Pierce LDH Cytotoxicity Assay Kit (Thermo scientific, Rockford, IL, USA). The release of LDH was measured at 490 nm by a Synergy HT plate reader (Biotech, Minneapolis, MN, USA) after a 6 h incubation.

### 2.10. Time-Kill Assay

Bacterial suspensions were prepared as described above. The killing kinetics assay of designed peptides was evaluated as described before, with a little modification [17]. Bacteria were incubated with a dose of peptides corresponding to 1 × MIC and 4 × MIC. The viable count was monitored for up to 24 h. Aliquots of 10 µl were taken at different times and appropriately diluted in PBS buffer (pH 7.4), and spread onto MHA plates. The number of CFU were counted after incubating at 37 °C overnight. Three experiments were run in triplicate.

### 2.11. Assessing Efficacy of Peptides Against MRSA In Vivo

This in vivo study of RPb was assessed on waxworms (*Galleria mellonella*) as the previous study with minor modifications [18]. 250 ± 25 mg of the waxworms (Livefood UK Ltd., Rooks Bridge, UK) were inoculated with 10 µL MRSA bacteria suspension in autoclaved saline (10^8^ CFU/mL). All the infected waxworms were confirmed to be alive at 2 h post-inoculation, followed by intrahaemocoelic injection of 20 mg/kg vancomycin (positive control group), 25 mg/kg peptides (sample group) and 50 mg/kg peptides (sample group). Saline and vancomycin (Sigma-Aldrich, Gillingham, UK) as negative control and positive control, respectively. Each group contained 9 waxworms and was repeated three times. All waxworms were observed every 12 h for 120 h.

### 2.12. Statistical Analysis

Data was analysed using Prism (Version 6.0; GraphPad Software Inc., San Diego, CA, USA). Error bars in the graphs represent standard error of the mean (SEM) with experiments performed on more than five replicates. Log-rank test was used to analyse the survival rate of waxworms. ∗ (*p* < 0.05), ∗∗ (*p* < 0.01) and ∗∗∗ (*p* < 0.001) represent significant difference.

## 3. Results

### 3.1. The Translated Open-Reading Frame Amino Acids Sequences of Ranatuerin-2 Peptides

**Figure 1:**
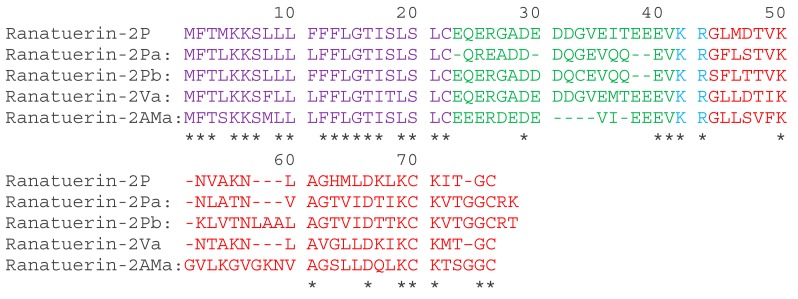
The translated open-reading frame amino acids sequences of ranatuerin-2 peptides from different species of frogs. The sequences of mature peptides in are labelled in red. The sequences of signal peptide are labelled in purple. Asterisks indicate the identical amino acid residues. The acid spacer peptide regions are labelled in green. The processing sites of the precursor for releasing mature peptides are labelled in blue. Gaps (dashed line) were introduced to optimise the identities.

A full-length cDNA, encoding ranatuerin-2Pb, was successfully cloned from the skin secretion –derived cDNA library of *Rana pipiens* (Figure S1). The alignments of amino acid sequences of selected ranatuerin precursors are shown in Figure 1. There were several typical structural characteristics, including a putative signal peptide region of 22 amino acid residues, an acidic spacer peptide region, a classical -KR- propeptide convertase processing site and a mature peptide of 34 amino acid residues. The nucleotide sequence of ranatuerin-2Pb precursor was deposited in the Genbank Nucleotide Sequence Database under the accession number MK922296. 

### 3.2. Peptide Design 

**Figure 2:**
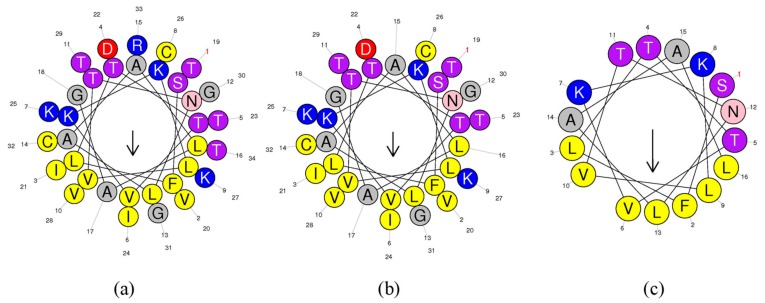
Heliquest of peptides. Heliquest of (**a**) ranatuerin-2Pb, (**b**) RPa and (**c**) RPb.

The helical wheel plots and secondary structures were predicted by online analysis tools. As shown in Figure 2, ranatuerin-2Pb and RPa shared a similar FVLGVIAVVLI hydrophobic face, however, RPb has less hydrophobic amino acid residues distributed on the hydrophobic face. Additionally, as shown in Table 1, ranatuerin-2Pb possessed +4 net charge with the hydrophobicity of 0.486 and the hydrophobic moment of 0.368; however, both RPa and RPb carried +3 net charge. Compared with ranatuerin-2Pb, RPa and RPb showed higher hydrophobicity which were 0.540 and 0.613, respectively (Table 1). All the peptides were purified by RP-HPLC and analysed by mass spectrometry (Figure S2).

### 3.3. Secondary Structure Analysis 

**Figure 3:**
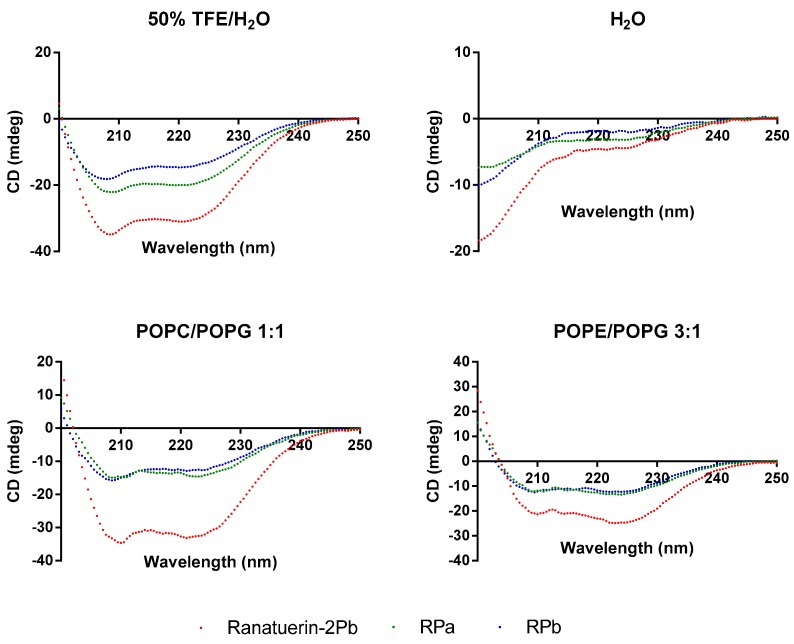
CD spectra record for Ranatuerin-2Pb, RPa and RPb in 50% TFE/H_2_O, H_2_O and LUV liposomes of *S. aureus* (POPC/POPG 1:1) and *E. coli* (POPE/POPG 3:1).

To demonstrate the changes of secondary structure of synthetic peptides in different environment, CD spectra was recorded in helical propagating solution (50% TFE/H_2_O), aqueous solution (H_2_O) and lipid bilayer liposomes of *S. aureus* (POPC/POPG 1:1) and *E. coli* (POPE/POPG 3:1) (Figure 3). All peptides were able to form α-helical structure in 50% TFE/H_2_O and membrane mimicking liposomes; however, the helical domain was eliminated in the aqueous environment. Based on the estimated secondary structure contents in Table 2, Ranatuerin-2Pb and RPb have similar helix proportion that are higher than RPa. Meanwhile, the helical content of Ranatuerin-2Pb was decreased when interacting with *E. coli* cell membrane-mimicking bilayer.

### 3.4. Antimicrobial and Haemolytic Activity of Peptides

**Figure 4:**
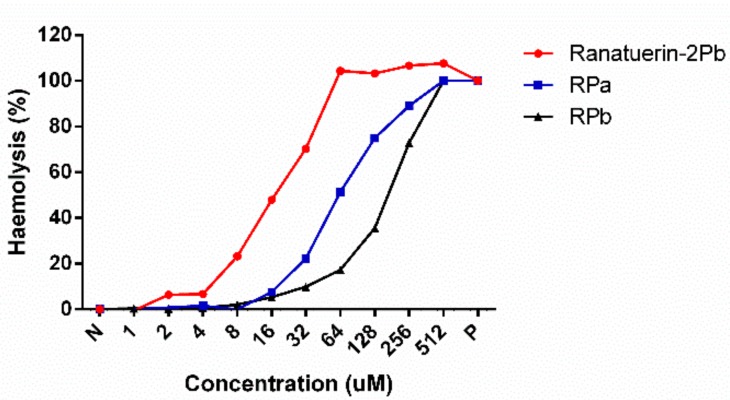
Haemolytic activities of Ranatuerin-2Pb, RPa and RPb. PBS and 1% TritonX100 were employed as negative control (N) and positive control (P), respectively. The 100% haemolysis was achieved by 1% TritonX100.

As shown in Table 3, ranatuerin-2Pb, RPa and RPb exhibited broad-spectrum antimicrobial activity. Specifically, ranatuerin-2Pb exhibited strong antimicrobial activity against *S. aureus*, *E. coli*, *C. albicans* and MRSA; however, it showed no inhibition activity against *E. faecalis* and *P. aeruginosa*. Additionally, RPa just possessed activity against *S. aureus* and *E. coli*. Notably, RPb exhibited significant broad-spectrum antimicrobial activity against all select tested microorganisms. Ranatuerin-2Pb, RPa and RPb exhibited a hemolysis rate near 20% on horse erythrocytes with the concentration at 8, 32 and 64 µM, respectively, as shown in Figure 4. In addition, The HC_50_ of ranatuerin-2Pb, RPa and RPb was 16.11, 63.90 and 178.0 µM, respectively. Notably, RPb exhibited the highest therapeutic index (TI) among the peptides.

### 3.5. Time-Kill Assay against S. aureus of Peptides

**Figure 5:**
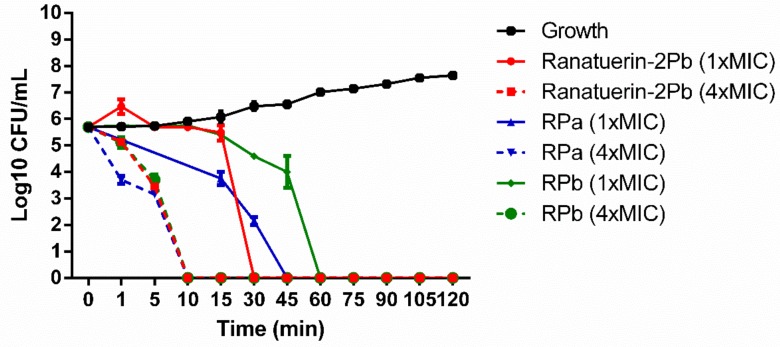
Time-killing curve of *S. aureus* by ranatuerin-2Pb, RPa and RPb at different concentrations. Growth control correspond to bacteria incubated in PBS without peptide. Data represent means ± SEM of three independent experiments.

The killing effect of peptides against *S. aureus* was evaluated at 1 × MIC and 4 × MIC of peptides. As shown in Figure 5. The time-killing curve revealed that ranatuerin-2Pb, RPa and RPb at 1 × MIC exerted killing activity at 30, 45 and 60 min while they were able to kill all bacteria at 10 min at their 4 × MIC. 

### 3.6. Antibiofilm Assay of Peptides against S. aureus

Ranatuerin-2Pb and RPb showed antibiofilm activity against *S. aureus*, *E. coli* and *C. albicans*. However, RPa just exhibited antibiofilm activity against *S. aureus* and *E. coli* biofilms. The minimum biofilm inhibitory concentration (MBIC) and the minimal biofilm eradication concentration (MBEC) of ranatuerin-2Pb, RPa and RPb against select tested bacteria biofilms are shown in Table 4.

### 3.7. MTT Cell Viability Assay

**Figure 6:**
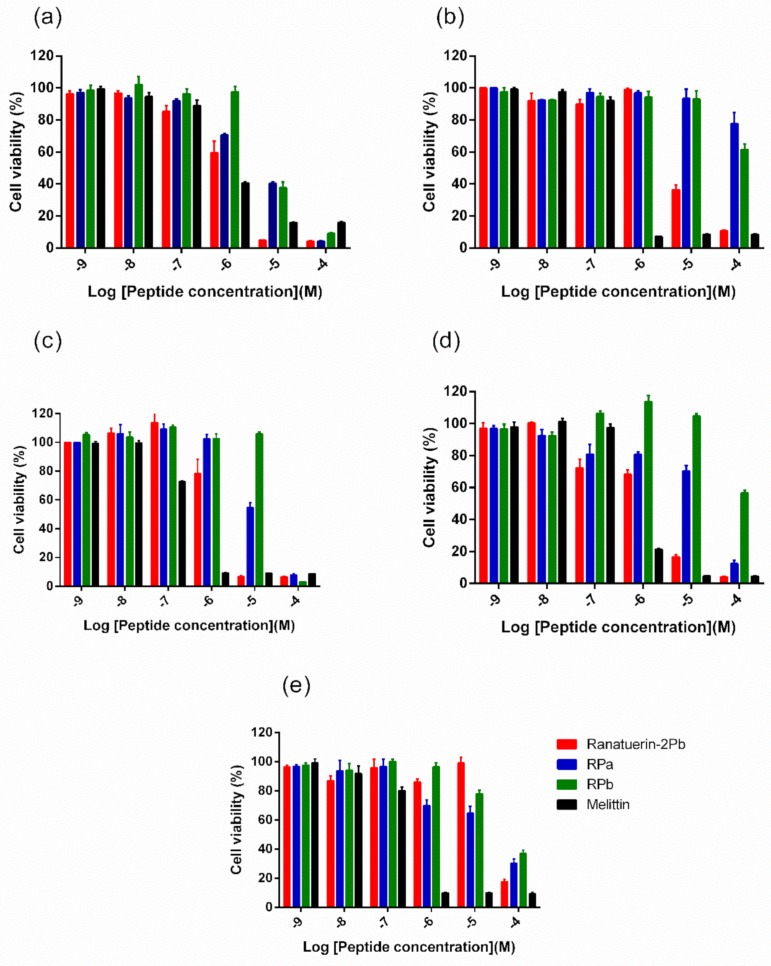
The effect on cell proliferation of peptides on the cancer cell lines (**a**) NCI-H157, (**b**) MCF-7, (**c**) U251MG, (**d**) PC-3 and (**e**) MDA-MB-435s. Ranatuerin-2Pb, RPa, RPb and Melittin are labelled red, blue, green and black, respectively. The 100% cell viability was applied with growing cells without peptide treatment. Data points represent the average of three independent experiments with error bars presenting the SEM.

Five human cancer cell lines were used to evaluate the anticancer ability of peptides. As shown in Figure 6, ranatuerin-2Pb exhibited significant inhibitory effect on the proliferation of NCI-H157, MCF-7, U251MG and PC-3, with the IC_50_ value of 1.453 µM, 7.254 µM, 2.172 µM and 2.251 µM, respectively. However, RPa and RPb just possessed inhibitory effect on the proliferation of H157; the IC_50_ of RPa and RPb was 5.841 µM and 6.856 µM, respectively. In addition, ranatuerin-2Pb, RPa and RPb showed no inhibitory effect on the proliferation of MDA-MB-435s.

### 3.8. LDH Assay

**Figure 7:**
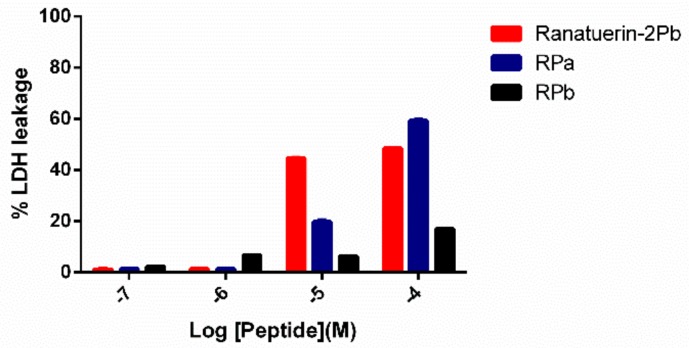
LDH leakage in H157 cells after 30 min of treatment with peptides at different concentrations in serum-free medium. Ranatuerin-2Pb, RPa and RPb are labelled as red, blue and black, respectively.

To further investigate the impact of ranatuerin-2Pb and its analogues on cancer viability, we examined their impact upon the lung cancer cell line NCI-H157 using the LDH assay. As shown in Figure 7, ranatuerin-2Pb induced the highest degree of LDH release for H157 among three peptides. However, RPb exhibited a negligible LDH leakage rate, which was consistent with the result of MTT assay. 

### 3.9. Membrane Permeability Assay

**Figure 8:**
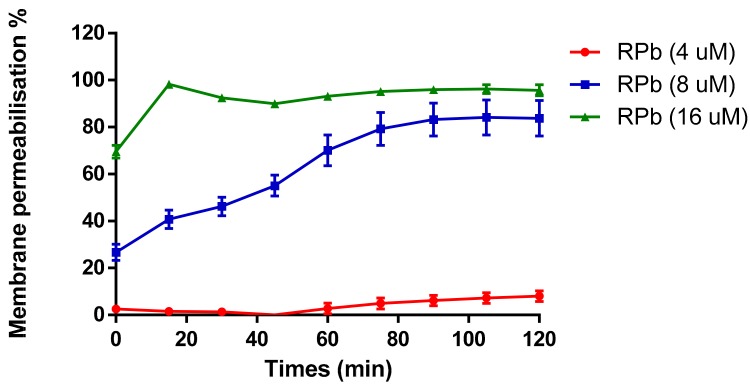
Membrane permeability against *S. aureus* with addition of different concentrations of RPb. The 100% cell membrane permeabilisation effect was performed using 8 µM of Melittin. Data are the means ± standard errors of the means (*n* = 3).

The cell membrane permeability of RPb was determined in a range of concentrations from 4 µM to 16 µM (Figure 8). It revealed that increasing the concentration of RPb induced the uptake of nucleus dye by *S. aureus*. With the increase of concentration of RPb, it induced significant and rapid membrane permeabilization. Moreover, 4×MIC of RPb resulted in an instant cell permeabilization effect that was similar to the effect of positive control, Melittin. 

### 3.10. Treatment of S. Aureus-Infected Waxworms with Peptides

**Figure 9:**
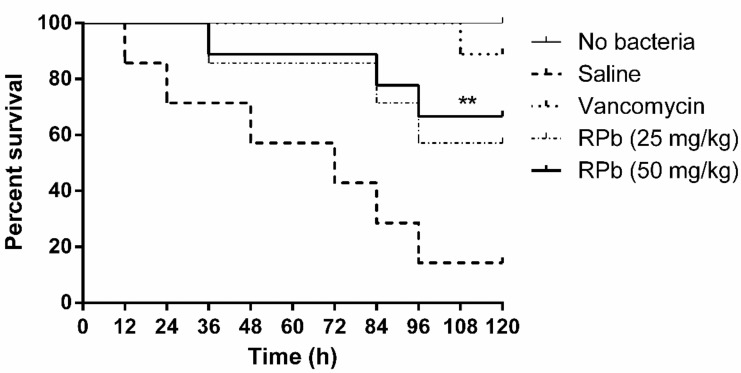
The percentage survival waxworms infected with MRSA. The infected waxworms were treated with vancomycin (20 mg/kg) and RPb (25 mg/kg and 50 mg/kg), respectively. ** *p* < 0.01 and * *p* < 0.05 indicate significant difference.

The mortality of *S. aureus*-infected waxworms after peptide treatment was significantly reduced as shown in Figure 9. Specifically, the survival rate of waxworms treated with 50 mg/kg of RPb was higher than that of waxworms treated with 25 mg/kg of RPb. In addition, RPb showed no cytotoxicity for waxworms.

## 4. Discussion

Ranatuerin-2 peptides have been found in most species of North American and Eurasian frogs [19], and it presented a less convincing phylogenetic marker than in the case of brevinin-1 and brevinin-2 [20]. Although ranatuerin-2Pb was previously identified in *Rana pipiens* [21], the cDNA encoding sequencing of this peptide was still unknown. Our study succeeded at the identification of the encoding gene of this skin defence peptide in the skin secretion of *Rana pipiens*. Recently, amounts of ranatuerin-2 peptides were isolated from skin secretions of *Lithobates*, *odorrana* and *rana* frogs [8]. Most ranatuerin-2 peptides possessed a broad-spectrum antimicrobial activity, though the primary sequences of these peptides are highly variable [20]. As shown in Figure 1, although there is a dramatic distinction in the length of mature peptide sequences and amino acid compositions, they still share common features, including the highly-conserved signal peptide sequence, the KR processing site and the C-terminal disulphate loop that exhibited unique sequence motif as -KCKXXGGC. 

Recently, various principles were adopted in the design of peptides, such as the replacement of specific amino acids and the truncation of original peptides [22,23]. As we knew, net charge, hydrophobicity, amphipathicity and α-helicity of antimicrobial peptides play significant roles in terms of increasing antimicrobial activity and reducing cytotoxic effects [24]. Those characteristics facilitate the mechanisms that AMPs electrostatically attached on the cell membrane and further interact with the lipid layer. Nevertheless, a clear explanation is that basic residues in peptides contribute to binding the phospholipid head groups and then the membrane morphology could be changed by the formation of transient transmembrane pores that small molecules like ions and ATP could leak out of the cell [25]. 

Herein, we observed that ranatuerin-2Pb exhibited nonspecific killing effect against bacterial cells, cancer cells and even red blood cells. Such manner was also reported in the case of the AMPs, like melittin that can induce the detergent-like effect upon the cell membrane [26]. To our understanding, eliminating the cytotoxicity without sacrificing biofunction could be achieved through optimisation of the ratio of these characteristics [27]. As our study showed, the removal of last two residues of ranatuerin-2Pb contributed to the decrease of the helical content for RPa that could explain the reduction of antimicrobial, anticancer and haemolytic activity for RPa compared to the parent peptide (Table 2). Although there is no NMR analysis performed for ranatuerin-2 peptides, the studies on Gaegurin, a 32-mer AMP from *Rana rugosa* possessing a C-terminal loop, illustrates that the structure is depicted by two locally well-defined helical domain helices, which are composed of residues 2–10 and 16–32 [28]. It suggests that ranatuerin-2Pb might also possess two helical segments at respective N-terminus and C-terminus, and the last two residues are probably included in the helix domain. Therefore, the removal of C-terminal residues results in the decrease of helical content that weakens the membrane permeabilisation. 

C-terminal “Rana box” is a unique structure among the AMPs discovered from Ranidae frogs. The function of the intramolecular disulphate bond has been considered to stably constrain the fold of C-terminal helical loop [29]. Functions of “Rana box” are remained ambiguous and controversial because the bioactivity of peptides changed or remained by removal of the loop were reported [30]. As our previous study demonstrated, the removal of C-terminal loop of nigrocin-HL increased the antimicrobial activity and, replaced by an amidated phenylalanine residue, resulted in the further enhancement of antimicrobial activity and reducing haemolysis activity [31]. Thus, further shortening the length of amino acids was performed. Notably, the helical content of RPb is around the similar proportion to RPa through the CD analysis (Table 2), which is consistent with our assumption about two helix segments located at both terminus of ranatuerin-2Pb. The N-terminal 16 amino acids could be the main contributors in the formation of α-helix at N-terminus. 

Interestingly, the 16-mer N-terminal segment, RPb, not only restored and broadened the antimicrobial activity but also reduced the toxicity to mammalian cells (Table 3 and Figure 6). Even more, the truncation slowed down the killing effect against *S. aureus*. It is reported that the two helical domains of Gaegurin induced a hinge region near Gly^24^, which the N-terminal helix could bend and insert into bilayer to form transmembrane pore while the C-terminal helix could attach on the lipid surface [29]. Such a feature may lead to the detergent-like effect at very low peptide-to-lipid ratios for membrane disintegration [32]. Also, the cytolytic peptide, Melittin, formed a hinge near Pro^14^ that results in two helical segments; however, the removal of the hinge domain significantly decreased the haemolysis [33]. The similar results were observed herein for RPb, indicating that the helix–kink–helix structure could improve the general membrane permeabilisation effect without specificity. RPb still retains the helical domain for insertion into the cell membrane, but it might need higher peptide-to-lipid ratios for inducing apparent membrane disintegration, which could be proven by the instant membrane permeabilisation caused by 16 µM of RPb (Figure 8). With regards to the reason of broad-spectrum activity of RPb, it is though unclarified, we assume that it would be influenced by the affinity towards different lipids [34]. Due to the helix–kink–helix conformation in ranatuerin-2Pb and RPa, they may be easy to diffuse across the peptidoglycan matrix of Gram-positive bacteria first and then act on the cytoplasmic membrane; however, they may be challenging to diffuse across the outer membranes of Gram-negative bacteria, which results in the loss of antimicrobial activity [35]. Taken together, our results revealed that a delicate balance of amphipathicity and α-helical is necessary for broad-spectrum antimicrobial activity and less toxicity.

Bacteria biofilms, the self-produced polysaccharide matrix that facilitates the adherence of microorganisms, provide better protection for bacteria, which can lead to antibiotic resistance in clinical infections [36]. In particular, sessile bacteria could tolerate much higher concentration of antimicrobial agents than the planktonic bacteria [37]. Our results demonstrated that ranatuerin-2Pb, RPa and RPb exhibited potent inhibition on the growth of biofilm against *S. aureus* (Table 3). Although the mechanism is unclear, a theory that the AMPs are transferred through the pores formed by the binding of the extracellular biofilm or only completely dispersed in the biofilm may explain this phenomenon [38]. 

Apart from antimicrobial activity, some AMPs also exhibited outstanding anticancer effects. Our results revealed ranatuerin-2Pb, RPa and RPb exhibited different anticancer effects on various cancer cells (Figure 6). Several theories have tried to explain the anticancer mechanism, including the interaction between AMPs and tumour cells, cell apoptosis, caspase activation, cytochrome C release, DNA fragmentation and mitochondrial membrane depolarisation [39,40,41,42]. However, based on our hypothesis above and the results of the LDH assay, the anticancer mechanism may mainly involve disruption of the cell membrane [41]. 

RPb exhibited higher therapeutic index, we therefore investigated the antimicrobial activity of RPb in the *S. aureus*-infected *Galleria mellonella* larvae model [43]. Although RPb shows a significant effect against *S. aureus* in vivo, the efficacy is still lower than that of the lead antibiotics. A similar study also reported that the antimicrobial potency was less than that of vancomycin in the treatment of MRSA infection [44]. It might be the nature of poor stability of AMPs in vivo. Further studies on peptide stability or even bioavailability should be taken into consideration. Additionally, RPb showed no acute toxicity in waxworms, which could provide a reference for further in vivo study. 

## 5. Conclusions

Ranatuerin-2Pb is an antimicrobial peptide that can form a helical structure to permeabilise bacterial cell membrane. It is potent against various microorganisms including the antibiotic-resistance strain, MRSA. However, the coherent helix–kink–helix structure may not be favourable to the selectivity towards bacteria and mammalian cell membrane. The truncated analogue, RPb, is able to enlarge the antimicrobial spectrum with the decrease of haemolytic effect. Although it exhibits significant antimicrobial activity both in vitro and in vivo, which warrants further investigation, detailed explanation of the mechanisms needs to be clarified. Overall, Ranatuerin-2Pb is a good antimicrobial template for further studying and developing new antimicrobial compounds. 

## Figures and Tables

**Table 1:** Physio-chemical properties of Ranatuerin-2Pb, RPa and RPb calculated by HeliQuest online tool.

Peptide	Sequence	H	µH	Net Charge (z)
Ranatuerin-2Pb	SFLTTVKKLVTNLAALAGTVIDTIKCKVTGGCRT-OH	0.486	0.368	4
RPa	SFLTTVKKLVTNLAALAGTVIDTIKCKVTGGC-OH	0.540	0.358	3
RPb	SFLTTVKKLVTNLAAL-NH_2_	0.613	0.511	3

**Table 2:** The estimated secondary structure content (%) of Ranatuerin-2Pb, RPa and RPb from obtained CD spectra in different solutions using BESTSEL CD spectrum analysis tool.

Solution	Peptide	Helix	Antiparallel	Parallel	Turn	Others
50% TFE/ H_2_O	Ranatuerin-2Pb	50	7.8	1.2	10.9	29.8
	RPa	33.6	18.1	4.3	11.4	32.5
	RPb	49.6	7.6	0	9.8	33
H_2_O	Ranatuerin-2Pb	3.5	26.6	0	18.3	51.5
	RPa	2.4	30.6	0	17.3	49.7
	RPb	0.3	25.4	0	19.4	55
POPC/POPG 1:1	Ranatuerin-2Pb	59.2	1.2	2.7	10.8	26.1
	RPa	24.9	13.7	8.6	13.7	39.1
	RPb	51.8	3.6	0	12.2	32.4
POPE/POPG 3:1	Ranatuerin-2Pb	40	3.8	8.7	11.8	35.7
	RPa	23.6	24.4	6.9	12.1	33
	RPb	51.6	9.2	0	12	27.2

**Table 3:** Minimum inhibition concentrations (MICs) (µM) and minimum bactericidal concentrations (MBCs) (µM) of peptides against tested bacterias.

		MICs/MBCs (µM)		
	Ampicillin	Ranatuerin-2Pb	RPa	RPb
*Staphylococcus aureus*	0.3/0.3	8/8	16/32	8/8
*Escherichia coli*	36.6/36.6	8/8	32/64	16/16
*Candida albicans*	146/>512	8/16	>256/>256	16/16
MRSA	>512/>512	16/32	>256/>256	16/32
*Enterococcus faecalis*	12.8/12.8	>256/>256	>256/>256	32/128
*Pseudomonas aeruginosa*	>512/>512	>256/>256	>256/>256	64/256
HC_50_	>512	16.11	63.90	178
TI (Overall)	19.42	0.449	0.353	8.83
TI (Gram-positive bacteria and yeast)	37.20	0.503	1.258	11.125

**Table 4:** Antibiofilm activity of peptides against tested bacteria.

	MBIC/MBEC (µM)		
	Ranatuerin-2Pb	RPa	RPb
*S. aureus*	8/32	16/128	8/32
*E. coli*	16/64	32/128	32/128
*C. albicans*	8/32	>256/>256	16/32

^1^ MBIC is the minimum biofilm inhibitory concentration. ^2^ MBEC is the minimum biofilm eradication concentration.

## Supplementary Materials

The following are available online at https://www.mdpi.com/2218-273X/9/6/249/s1, Figure S1. Nucleotide and corresponding translated open-reading frame of precursor cDNA cloned from the skin secretion of Chinese piebald odorous frog cDNA library that encodes ranatuerin-2Pb. Figure S2. RP-HPLC chromatogram and mass spectrums of synthesised peptides, ranatuerin-2Pb (a), RPa (b) and RPb (c).
