# Supplementary material for: Bioevaluation of Ranatuerin-2Pb from the Frog Skin Secretion of Rana pipiens and Its Truncated Analogues
Source: Biomolecules. 2019 Jun 25;9(6):249. doi: 10.3390/biom9060249 (PMC6627226; doi:10.3390/biom9060249)
Supplement: Supplementary file 1 [file biomolecules-09-00249-s001.pdf]

Bio-evaluation of Ranatuerin-2Pb from the Frog Skin Secretion of *Rana Pipiens* and its Truncated Analogues

Supplement materials:

```
    M  F  T  L   K  K  S   L  L  L   F  F  F  L   G  T  I ·
  1 ATGTTCACCT TGAAGAAATC CCTGTTACTC TTTTTCTTTC TTGGGACCAT
    TACAAGTGGA ACTTCTTTAG GGACAATGAG AAAAAGAAAG AACCCTGGTA
    · S  L  S   L  C  E  Q   E  R  G   A  D  D   D  Q  G  E
 51 CTCCTTATCT CTCTGTGAGC AAGAGAGAGG TGCCGATGAC GACCAAGGGG
    GAGGAATAGA GAGACACTCG TTCTCTCTCC ACGGCTACTG CTGGTTCCCC
    ·  V  Q  Q   E  V  K   R  S  F  L   T  T  V   K  K  L
101 AAGTACAGCA AGAAGTAAAA AGAAGTTTCC TTACTACAGT CAAGAAGTTG
    TTCATGTCGT TCTTCATTTT TCTTCAAAGG AATGATGTCA GTTCTTCAAC
    V  T  N  L   A  A  L   A  G  T   V  I  D  T   I  K  C ·
151 GTTACCAATC TGGCAGCACT GGCAGGGACT GTGATAGATA CCATAAAATG
    CAATGGTTAG ACCGTCGTGA CCGTCCCTGA CACTATCTAT GGTATTTTAC
    · K  V  T   G  G  C  R   T  *
201 TAAAGTTACT GGAGGATGTA GAACGTGAAT TGGAAGTCAT CTGATGTGGC
    ATTTCAATGA CCTCCTACAT CTTGCACTTA ACCTTCAGTA GACTACACCG
251 GTTCATTTAG CTAAATGCTA CATGTCTAAT AAAAAATACA AATTTCACAA
    CAAGTAAATC GATTTACGAT GTACAGATTA TTTTTTATGT TTAAAGTGTT
301 AAAAAAAAAA AAAAAAA
    TTTTTTTTTT TTTTTTT
```

Figure S1. Nucleotide and corresponding translated open-reading frame of precursor cDNA cloned from the skin secretion of Chinese piebald odorous frog cDNA library that encodes ranatuerin-2Pb. Putative signal peptide is double-underlined, mature peptide is single-underlined and stop codon is marked by asterisk.

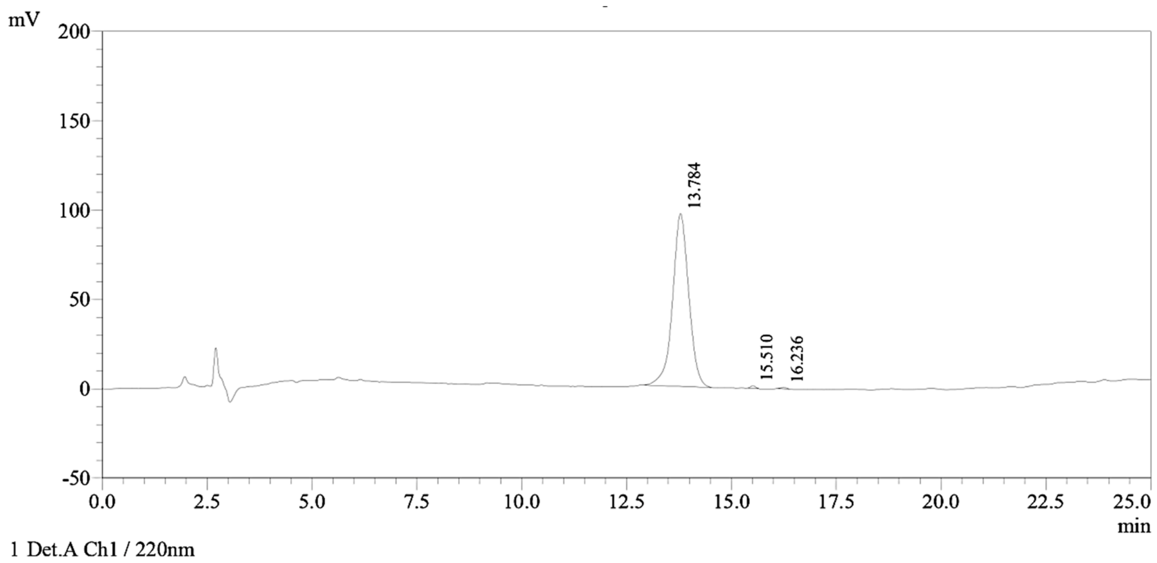

Peak Table

Detector A Ch1 220nm

Peak#	Ret. Time	Area	Height	Area %
1	13.784	2600668	96731	99.271
2	15.510	12142	1449	0.463
3	16.236	6945	783	0.265
Total		2619755	98963	100.000

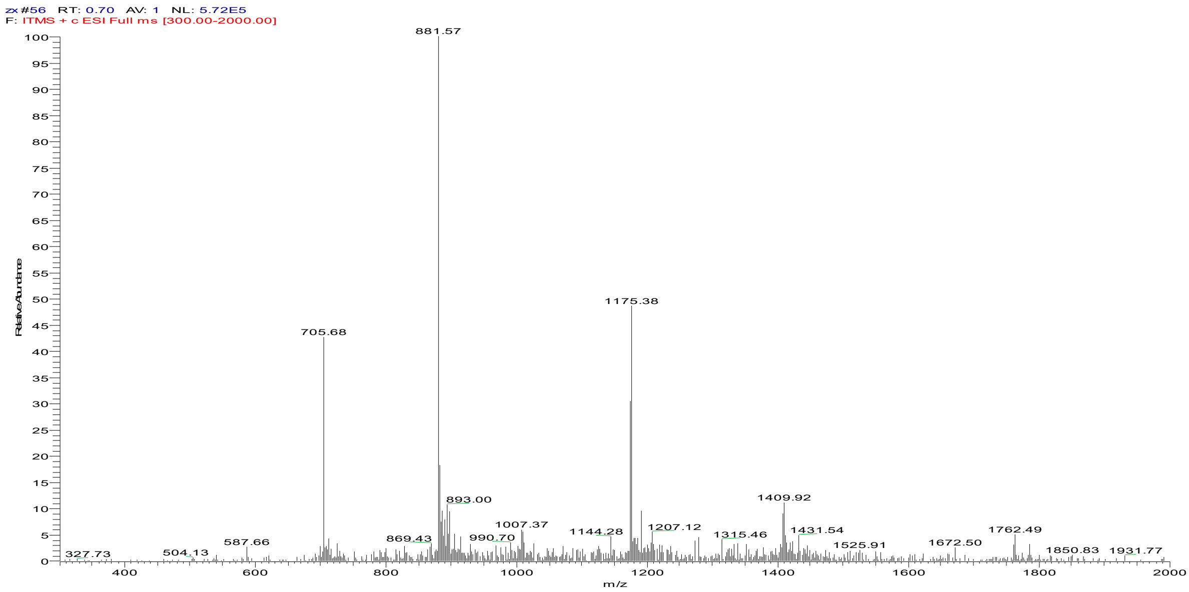

(a)

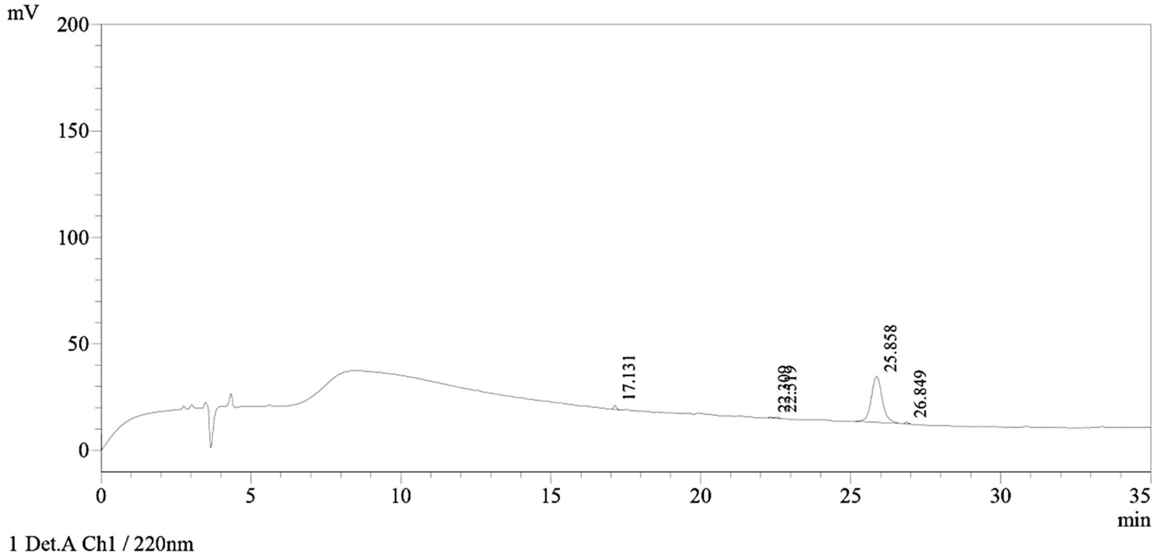

1 Det.A Ch1 / 220nm

Peak Table

Detector A Ch1 220nm

Peak#	Ret. Time	Area	Height	Area %
1	17.131	12570	1835	2.195
2	22.309	3632	459	0.634
3	22.519	4692	505	0.819
4	25.858	545334	21581	95.228
5	26.849	6436	824	1.124
Total		572664	25205	100.000

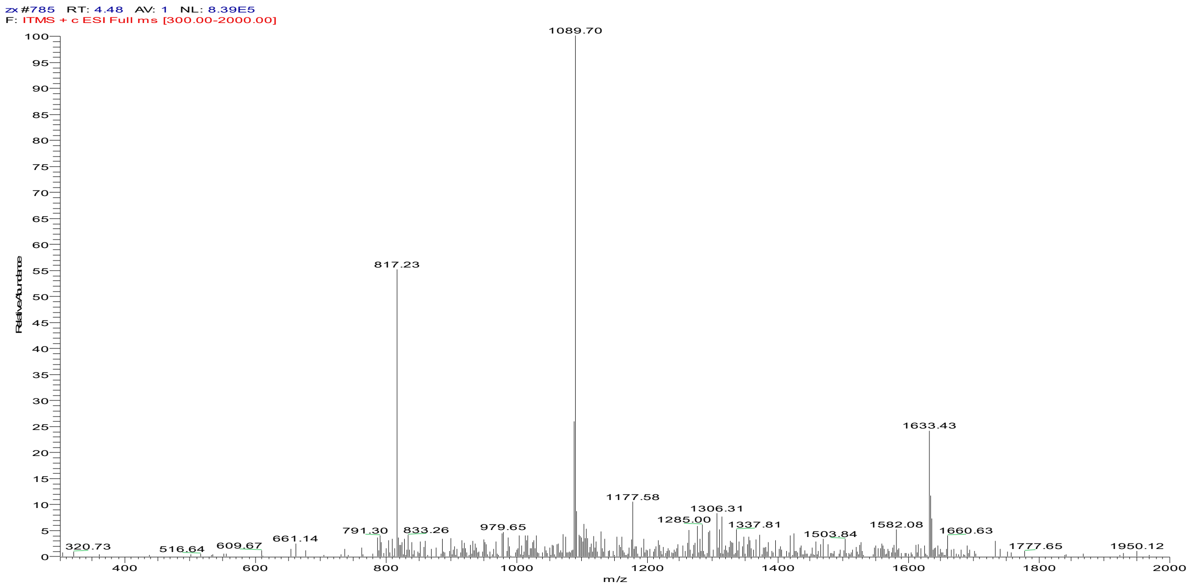

(b)

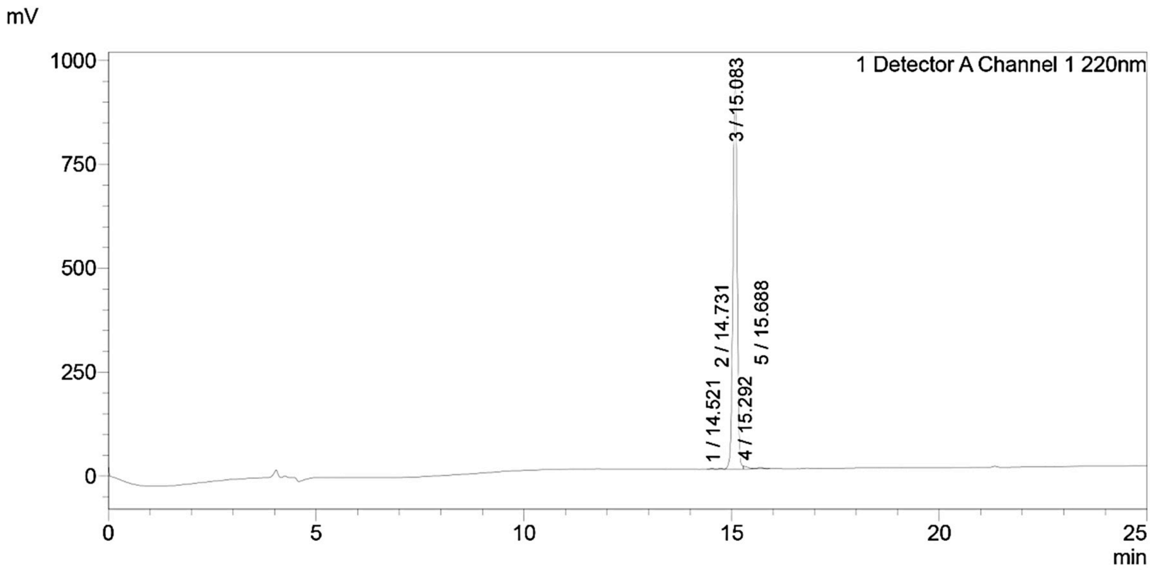

<Peak Table>

Detector A Channel 1 220nm

Peak#	Ret. Time	Area	Height	Area%
1	14.521	11565	2106	0.172
2	14.731	12738	2125	0.189
3	15.083	6632957	947951	98.515
4	15.292	45654	6695	0.678
5	15.688	29999	3834	0.446
Total		6732913	962711	100.000

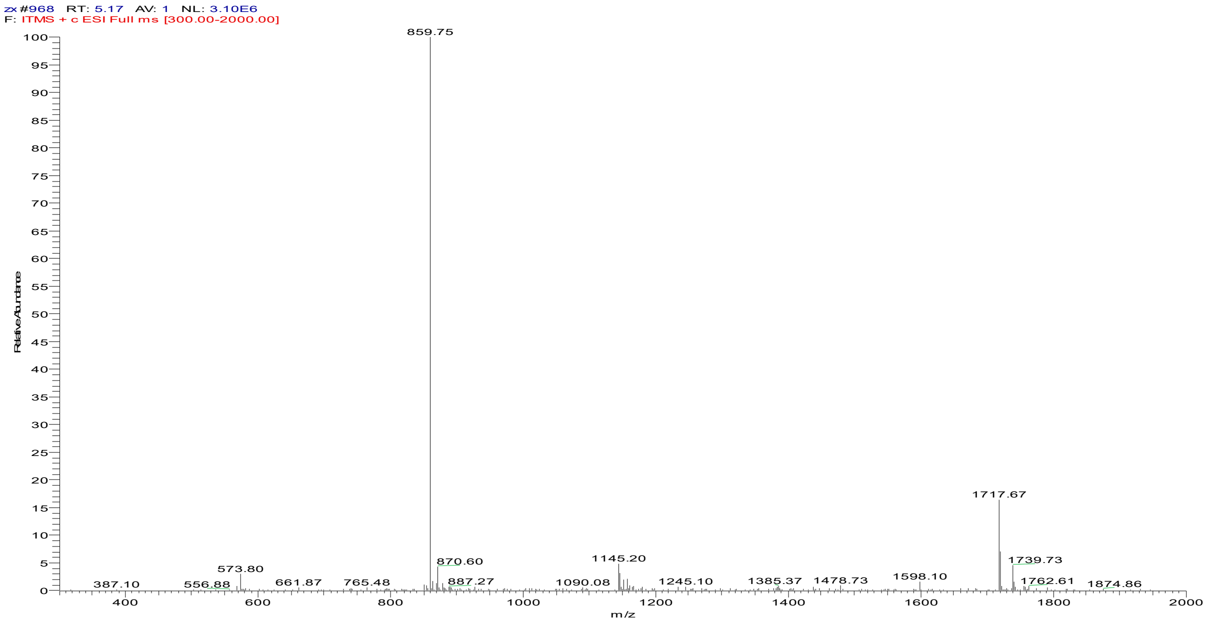

(c)

Figure S2. RP-HPLC chromatogram and mass spectrums of synthesised peptides, ranatuerin-2Pb (a), RPa (b) and RPb (c).
